# Chestnut Shell-Activated Carbon Mixed with Pyrolytic Snail Shells for Methylene Blue Adsorption

**DOI:** 10.3390/ma15228227

**Published:** 2022-11-19

**Authors:** Jiahao An, Nguyen Thi Hong Nhung, Yaxuan Ding, Hao Chen, Chunlin He, Xinpeng Wang, Toyohisa Fujita

**Affiliations:** 1 School of Resources, Environment and Materials, Guangxi University, Nanning 530004, China; 2 School of Chemistry and Chemical Engineering, Guangxi University, Nanning 530004, China

**Keywords:** snail shells, chestnut, pyrolysis, adsorption, methylene blue

## Abstract

Activated carbon has been used to treat organic dyes in water systems; however, the adsorption capacity of the samples studied was limited by the specific surface area and influenced by the pH of the aqueous solution. In this study, a hybrid adsorbent consisting of a mixture (MCS) of activated chestnut shell biochar (CN) and pyrolyzed snail shell material (SS) was developed to solve this problem, with the waste snail shell samples being processed by pyrolysis and the chestnut shell samples chemically pretreated and then pyrolyzed. The BET and SEM results revealed that the SS had a mesoporous fluffy structure with a higher specific surface (1705 m^2^/g) and an average pore diameter of about 4.07 nm, providing a large number of sites for adsorption. In addition, XPS and FTIR results showed that the main component of SS was calcium oxide, and it also contained a certain amount of calcium carbonate, which not only provided an alkaline environment for the adsorption of biochar but also degradation and photocatalytic capabilities. The results showed that the MCS3-1 sample, obtained when CN and SS were mixed in the ratio of 3:1, had good capacity for adsorption for methylene blue (MB), with 1145 mg/g at an initial concentration of 1300 mg/L (92% removal rate). The adsorption behaviors were fitted with the pseudo-second-order kinetic model and Freundlich isotherm model, which indicated that the adsorption was multilayer chemisorption with a saturated adsorption capacity of 1635 mg/g. The photocatalytic capacity from the SS composition was about 89 mg/g, and the sorption of MB dye onto the sorbent reached equilibrium after 300 min. The results suggested that MCS3-1 has enormous potential for removing MB from wastewater.

## 1. Introduction

Water is a necessity for all forms of life. However, due to the increasing global population, urbanization, and industrialization, water resources are under serious threat [1]. Methylene blue (MB) is a cationic dye widely used in the dyeing, textile, leather processing, pulp, and paper industries, as well as other fields [2]. It is composed of methylthioninium chloride and is widely used as a synthetic dye due to its high solubility and color stability. However, it has been found that only 5% of the dye is used during the dyeing process with the remainder discharged [3]. Due to its inherent characteristics of being difficult to degrade, toxic, and water-soluble, it aggravates water pollution, causes ecological damage, and threatens human health [4,5]. In recent years, various physical, chemical, and biological methods have been used for decontamination of dyestuffs. Current treatment methods include adsorption [6], coagulation [7], the membrane process, electrochemical separation, biological treatment, and catalysis [8]. Among these methods, adsorption technology and photocatalysis technology offer the advantages of high efficiency, safety, and low cost, and the raw materials used are readily available [9,10].

Biochar is a kind of carbon material obtained through high-temperature and oxygen-free atmosphere pyrolysis using biomass as a precursor [11]. Activated carbon materials can be obtained through physical or chemical activation of biomass, which has the advantages of having high specific surface area and abundant functional groups and producing no environmental hazards. Therefore, there is a need to find a reliable source of carbon that is both cheap and easily accessible for biochar production. Many cash crops have been targeted by researchers, such as rice husks [12], coconut husks [13], corn cobs [14], tapioca peel [15], walnut shells [16], sugarcane bagasse [17], and tea factory waste [18]. Chestnuts are one such material, and they are an economic forest species in China, making the effective utilization of chestnut shells a huge market prospect. Unlike the powder structures obtained by crushing other raw materials, crushing chestnut shells results in a loose, cotton-like fiber mass [19], as the shells themselves are composed of a fibrous arrangement, which facilitates the penetration of reagents into the chestnut shells during chemical activation, allowing the raw material to be fully activated. In previous studies, the use of activated carbon for MB adsorption required an alkaline environment to increase the adsorption capacity. Sodium hydroxide (NaOH) has usually been used to adjust the pH of the solution to achieve suitable adsorption conditions, but this drives up the cost of disposal even further and consumes a large amount of alkalis [5,15,20]. Therefore, a low-cost and easily available source of alkalis is also required.

Calcium carbonate (CaCO_3_), which decomposes to calcium oxide and carbon dioxide at high temperatures, is the main component of the shells. In other studies, snail shell-derived biochar has been used for the removal of heavy metal ions through ion exchange [21], and organic dyes have been removed from wastewater by taking advantage of the adsorption capacity [22] and photocatalytic degradation [23,24]. In this study, a mixture of high-temperature calcined *Oncomelania hupensis Gredler* shell-derived biochar (SS) and chestnut shell-derived carbon (CN) was used as an adsorbent to remove MB. The calcium oxide of SS, which was produced by calcinating the shells at high temperatures, dissolving them, and hydrolyzing them in water to produce hydroxide, served to provide an alkaline environment for the adsorption of CN and enhanced its adsorption capacity. In addition, CaO not only provided an alkali environment for the adsorption of activated carbon but could also degrade MB through photocatalysis [25]. Therefore, a mixture of SS and CN was used for MB-adsorption photocatalytic removal. The ability of the mixture to remove MB aqueous solution was characterized. The effects of several parameters, including the solution pH, dye concentration, sorbent dose, and ratio, were studied. The result demonstrated that the mixture efficiently removed MB from water.

## 2. Materials and Methods

### 2.1. Material and Reagents

All the chemicals and reagents were of analytical grade and used without further purification. Chestnut and *Oncomelania hupensis Gredler* snails were bought in a local market (Nanning, China), and hydrochloric acid (HCl, 36~38%, analytical reagent), nitric acid (HNO_3_, 65~68%, analytical reagent), sulfuric acid (H_2_SO_4_, 95~98%, analytical reagent), sodium hydroxide (NaOH, analytical reagent), phosphoric acid (H_3_PO_4_, ≥85%, analytical reagent), and methylene blue (analytical reagent) were purchased from Sinopharm Chemical Reagent Beijing Co, Ltd. Deionized water of 18.2 MΩ⋅cm was used in preparing the solutions and testing.

### 2.2. Preparation of the Adsorbent and MB Solution

The pulp from the snails and chestnuts was washed three times in deionized water, and shells were dried in an oven at 80 °C for 48 h. The pretreated chestnut shells and snail shells were cooled to room temperature. Chestnut shells were broken into small pieces.

To activate the chestnut shells, 20 g of pretreated chestnut shells was soaked in 80 mL of 40% phosphoric acid for 24 h before being dried in the oven for 48 h. The sample was then pyrolyzed in a muffle furnace at 450 °C under a nitrogen atmosphere for 2.5 h. Then, the chestnut shell-activated carbon was thoroughly washed with deionized water until a neutral pH was obtained and dried in an oven. The dried products were crushed and sieved with an 80–200 mesh sieve for adsorption experiments. The material obtained was named CN.

The pretreated snail shells were pyrolyzed in a muffle furnace at 500, 700, and 900 °C under a nitrogen atmosphere for 1 h. The samples obtained were crushed into powder and named SS-500, SS-700, and SS-900 according to the pyrolysis temperature.

The mixture of CN and SS was named MCS. The adsorbent samples with different mixing ratios were named SS, MCS1-3, MCS2-2, MCS3-1, and CN (20 mg; CN to SS: 1:3, 1:1, and 3:1, respectively).

The MB solution was prepared by weighing the corresponding mass of the MB solid in a volumetric flask and fixing the volume with deionized water; for example, 1000 mg/L of MB solution was obtained by fixing 1000 mg of MB solid in a 1 L volumetric flask.

### 2.3. Materials Characterization

The characteristics of CN and SS were studied separately due to the simple physical mixing and assessed with thermogravimetric analysis (TGA), X-ray diffraction (XRD), Fourier-transform infrared spectroscopy (FT–IR), scanning electron microscopy (SEM) and nitrogen adsorption isotherms using the Brunauer, Emmet, and Teller (BET) method, and X-ray photoelectron spectroscopy (XPS).

The TGA-DSC analyses were performed with Netzsch equipment, model STA 449 F3 Jupiter (Germany), under a nitrogen gas flow of 50 mL/min and at temperature ranges of 30–600 °C and a heating rate of 5 °C/min and 30~1000 °C and a heating rate of 10 °C/min for chestnut and snail shells, respectively. The specific surface area and porosity of the samples were analyzed using N_2_ adsorption–desorption experiments at 423.15 K over 12 h in a vacuum before the test (Ttistarll 3020, Micromeritics Instrument Corp., Norcross, GA, USA), and the specific surface area of external pores was determined with the BET method. The surface charge of biochar was measured with a zeta potential analyzer (Nanobrook Omni, Brookhaven, New York, NY, USA). High-resolution field emission SEM (Sigma 300 Zeiss, Oberkochen, Germany) with an acceleration of 5 kV without gold-coating treatment was used to study the surface structure of the material. X-ray photoelectron spectroscopy (Smarelab-3 kV, Rigaku, Japan) was used to characterize the changes in elemental chemical states on the surface of the adsorbent before and after adsorption. The crystalline structure of the material was characterized with XRD, with a Cu K-β line (40 kV, 30 mA, 2θ = 10–80°) as the radiation source. Fourier-transform infrared spectroscopy (Shimadzu IRTracer-100, Shimadzu Co., Kyoto, Japan) was used to characterize the functional group changes before and after pyrolysis and the adsorption process in the range from 400 to 4400 cm^−1^. All concentrations were measured with UV spectroscopy at 665 nm, which is the maximum absorption wavelength of MB.

#### 2.3.1. Dye Adsorptive Experiments

MB aqueous solution was prepared using different masses of MB with ultrapure water, and the concentration of this solution was determined with a UV spectrophotometer at 665 nm. Sample bottles containing sorbents and MB were shaken at 298.2 K using a shaker at an oscillation frequency of 140 rpm for the period until the solution reached equilibrium or the adsorption process was completed. The effects of the pH, adsorbent ratio, initial concentration, and contact time of the dye solution were analyzed using a single-factor test. Different pH values (from 4 to 12) for the solution were investigated to analyze the effects of solution acidity and basicity on the adsorption results. Under the same conditions, the pH of the solution was increased from 4 to 12 using 0.1 M HCl and 0.1 M NaOH. The effect of the adsorbent ratio on adsorption was investigated by adding equal amounts of adsorbents with different ratios (20 mg; 0:4, 1:3, 1:1, 3:1, 4:0) to solutions of the same concentration and, after adsorption, the samples were placed in an incubator for 24 h with simulated daylight exposure to reveal the effect of ratio on the photocatalytic capacity. The effect of the initial concentration was studied by changing the concentration of the solution (500, 900, 1300, 1700, 1900, and 2100 mg/L) and, after adsorption, samples were placed in an incubator for 24 h to reveal the effect of ratio on photocatalytic capacity. To further investigate the effect of contact time on the adsorption capacity, the stirring times were varied to 0.25, 0.5, 1, 2, 3, 5, 10, and 24 h, respectively.

The MB concentration at this point was measured using UV–Vis, as it is a simple method for detecting the concentration of organic dyes at 665 nm, which is the maximum absorbance of MB. The removal percentage (*RM*%) of MB by the adsorbent was calculated with Equation (1):

(1) $$\mathrm{RM}\;\%=\frac{C_{0}-C_{e}}{C_{0}}\;\times \;100\%$$

where *C*_0_ (mg·L^−1^) and *C_e_* (mg·L^−1^) represent the initial and equilibrium MB concentrations, respectively.

The adsorbent’s adsorption capacity for MB was calculated with Equation (2):

(2) $$q_{e}=\frac{\left(C_{0}-C_{e}\right)V}{W}\;$$

where *q_e_* (mg·g^−1^) is the adsorption capacity of the adsorbent, *V* (L) represents the volume of the dye solution, and *W* (g) is the adsorbent weight.

The above equations were used to calculate the relationship between adsorbent, adsorption capacity, and removal rate. To further understand the intrinsic mechanism of the adsorption process, kinetic and isothermal studies were used and the results fitted to different models to determine the actual situation of the adsorption.

#### 2.3.2. Equilibrium Isotherm Studies

The equilibrium isotherm is critical for understanding the nature of the specific relationship between adsorbate molecules and an adsorbent [6]. The adsorption isotherms were determined using 20 mg of the selected samples in 20 mL of the dye solutions at different concentrations. After the adsorption process, the supernatant was used to determine the concentration at the equilibrium condition (*C_e_*). The isotherm studies were represented using Langmuir and Freundlich adsorption isotherm models. The Langmuir isotherm is assumed to represent monolayer adsorption [26,27], and adsorption can only occur in a limited number of identical and equivalent local locations, as shown in Equation (3):

(3) $$\frac{C_{e}}{q_{e}}=\frac{1}{q_{m}}C_{e}+\frac{1}{q_{m}k_{L}}$$

where *q_m_* (mg·g^−1^) and *q_e_* (mg·g^−1^) are the maximum adsorption capacity and equilibrium adsorption capacity of the adsorbent, respectively, and *k_L_* (mg·L^−1^) is the Langmuir constant related to the adsorption capacity and the rate of adsorption.

The Freundlich isotherm is an empirical model used to describe a heterogeneous surface [28]. The model assumes that the adsorbent sites have different adsorption energies. The Freundlich isotherm is calculated with Equation (4)

(4) $$\mathrm{lg}q_{e}=\mathrm{lg}K_{F}+\frac{1}{n}\mathrm{lg}C_{e}$$

where *K_F_* represents the adsorption rate and 1/*n* represents the adsorption strength. *K_F_* and 1/*n* can be determined from a linear plot of lg*q_e_* and lg*C_e_*.

#### 2.3.3. Adsorption Kinetics Studies

To investigate the adsorption kinetics, pseudo-first-order (PFO) kinetic models and pseudo-second-order (PSO) kinetic models were used to probe the capacity for the adsorption of MB onto each adsorbent surface. The PFO model relates the adsorption rate to the number of vacant sites on the adsorbent. This process is a diffusion-dependent one that is termed physisorption because it uses a physical process to remove dye from an aqueous solution. The van der Waals force and mechanical adhesion are involved [29,30]. When adsorption involves diffusion across a boundary, most systems follow the pseudo-first-order kinetic model calculated with Equation (5)

(5) $$q_{t}=q_{e}\left(1-e^{-k_{i}t}\right)$$

where *k*_1_ (min^−1^) denotes the adsorption rate constant. The pseudo-second-order kinetic equation reveals the behavior over the entire adsorption range. Chemisorption is a rate-control step calculated with Equation (6):

(6) $$q_{t}=\frac{k_{2}q_{e}^{2}t}{1+k_{2}q_{e}t}$$

where *k*_2_ (g·mg^−1^·min^−1^) denotes the adsorption rate constant of the pseudo-second-order equation, which reflects the speed of the adsorption rate, and, in both equations, *q_t_* (mg·g^−1^) and *q_e_* (mg·g^−1^) stand for the values for adsorption performance.

#### 2.3.4. Desorption Studies

The desorption experiment used the dried MCS3-1 sample at the end of adsorption, which was quantitatively weighed and placed in 0.1 M HCl, 0.1 M HNO_3_, 0.1 M H_2_SO_4_, 0.1 M NaOH, and pure water for desorption. The MB concentration in the solution was measured after shaking at 140 RPM for 8 h in a shaker.

## 3. Results and Discussions

### 3.1. Characterization of the Adsorbent

#### 3.1.1. TGA-DSC Analysis

**Figure 1:**
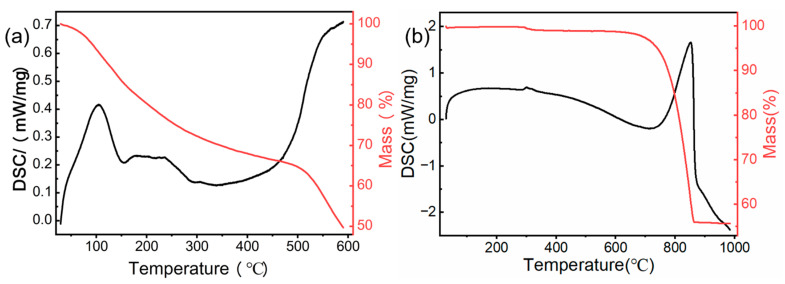
TGA and DSC curves of (**a**) chestnut shells and (**b**) snail shells.

Figure 1a shows the TGA-DCS results for the chemically pretreated chestnut shells. At the beginning of the TGA curve, the loss of mass at 50~200 °C was due to the evaporation of moisture from the material and the decomposition of volatile organic compounds [31]. In the region where the temperature rises from 200 to 500 °C, a slow weight-loss process occurred, which was the pyrolysis of the cellulose and hemicellulose of the chestnut shells. Further rapid weight loss occurred above 500 °C due to the pyrolysis of the relatively high proportion of lignin in the chestnut shells [32], and the same result is shown in the DSC curve. To avoid damage to and loss of the pore structure in the material, the chestnut shells were pyrolyzed at 450 °C.

The curves for the snail shell sample are shown in Figure 1b. Since it was fully dried in the oven before pyrolysis, the moisture in the material had been eliminated. Thus, the mass loss was divided into two stages. At stage one, a tiny weight loss phase occurred due to the organic matter decomposition at approximately 300 °C. At 600~800 °C, significant weight loss could be observed due to the degradation of the calcium carbonate at high temperatures. The chemical reaction is shown in Equation (7). In this stage, approximately 40% of the mass of the raw material was lost:

(7) $${\mathrm{CaCO}}_{3}\;\overset{\;800\;{}^{\circ}C}{\to }{\mathrm{CaO}+\mathrm{CO}}_{2}$$

Based on the above results, we chose to pyrolyze the chemically treated chestnut shells at 450 °C for 2.5 h with a heating rate of 5 °C min^−1^ and the snail shells at 900 °C for 1 h with a heating rate of 10 °C min^−1^, both under nitrogen atmosphere.

#### 3.1.2. XRD Analysis

**Figure 2:**
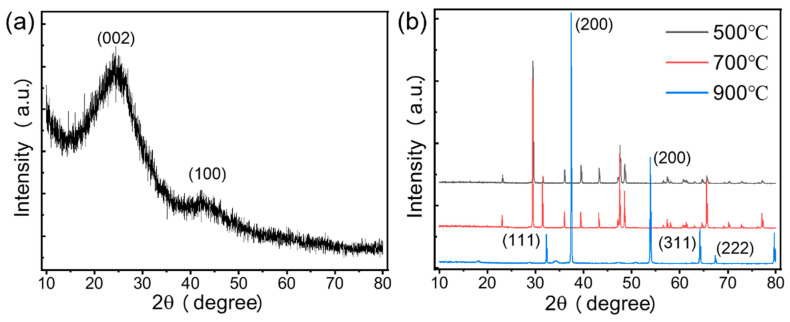
XRD analysis of (**a**) CN and (**b**) SS at 500, 700, and 900 °C.

The XRD analyses of CN and SS were performed separately because the mixed samples were simply physically mixed and no changes in the crystal structure of the materials occurred. Figure 2a shows the results for the CN sample, there are two peaks in this figure at the 2θ values of 23.72° and 42.32°, which belong to the (002)-type crystalline plane and (100)-type crystalline plane, respectively [33,34]. The presence of both graphite carbon peaks indicates that the material was partially graphitized during pyrolysis, but the main structure remained amorphous carbon. There was no significant difference in the peak positions compared to other carbon materials pretreated with different chemical reagents [35,36,37]. This indicated that phosphoric acid mainly affected the organic composition and pore structure of the material surface but not the crystalline structure.

Figure 2b shows the XRD curves for snail shells obtained with pyrolysis at three different temperatures (500, 700, and 900 °C). Under 500 and 700 °C pyrolysis conditions, the peak positions of the samples SS-500 and SS-700 were almost the same, and the main components in both materials were CaCO_3_. However, after increasing the pyrolysis temperature to 900 °C, major peaks at the 2θ values of 32.26, 37.42, 53.88, 64.18, and 67.38° were observed, which were similar to CaO peaks with h k l planes of 32° (1 1 1), 37° (2 0 0), 54° (2 2 0), 64° (3 1 1), and 67° (2 2 2) [25,38]. This indicated that the CaCO_3_ component of the snail shells decomposed and was converted to CaO. The above findings indicated that 900 °C was the best pyrolysis condition to obtain the CaO material.

#### 3.1.3. SEM-EDS Analysis

**Figure 3:**
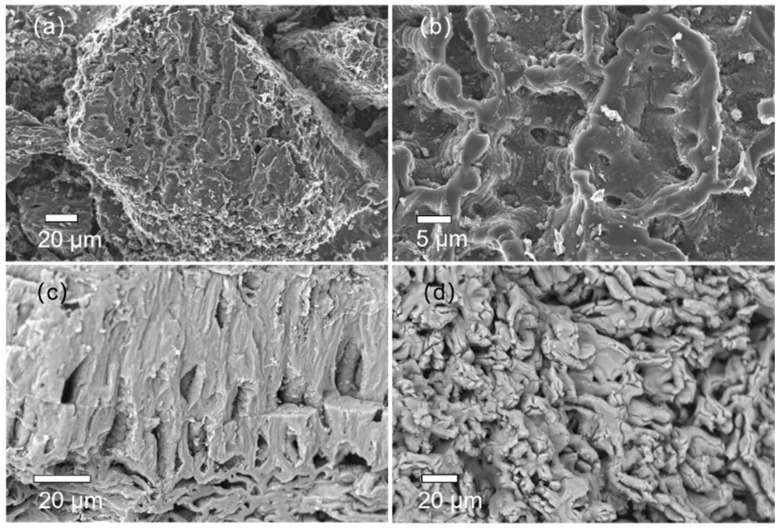
SEM images of (**a**) CN (500×), (**b**) CN (2000×), (**c**) MCS3-1, and (**d**) SS.

The SEM of the surface structure of the sample at different magnifications is shown in Figure 3. The SEM image in Figure 3a shows that the surface of the material was very rough with undulating layers of a range-like structure, and there were many defective pores. This means that the material had a large specific surface area that could provide a large number of adsorption sites. Another reason for this porous structure was that, under high-temperature anaerobic conditions, phosphoric acid and its derived polyphosphate esters combine with organic functional groups in the raw material with the effect of helping pore formation, as shown by Equation (8):

(8) $$4H_{3}{\mathrm{PO}}_{4}+10C\to P_{4}+10\mathrm{CO}+6H_{2}O$$

During the pyrolysis process, phosphoric acid combines with carbon to form carbon monoxide that escapes from the material. This resulted in the ablation of the carbon and the production of pore structures where carbon was lost.

An SEM image of the structure of the mixture sample after adsorption is shown in Figure 3c. The surface of CN remained rough, with a laminar structure and pores between the layers. In addition, some tiny pore structures on the surface of the structure disappeared due to MB adsorption and Ca(OH)_2_ precipitation on the surface. Figure 3d presents an SEM image of the surface structure of SS, showing scale-like structures.

**Figure 4:**
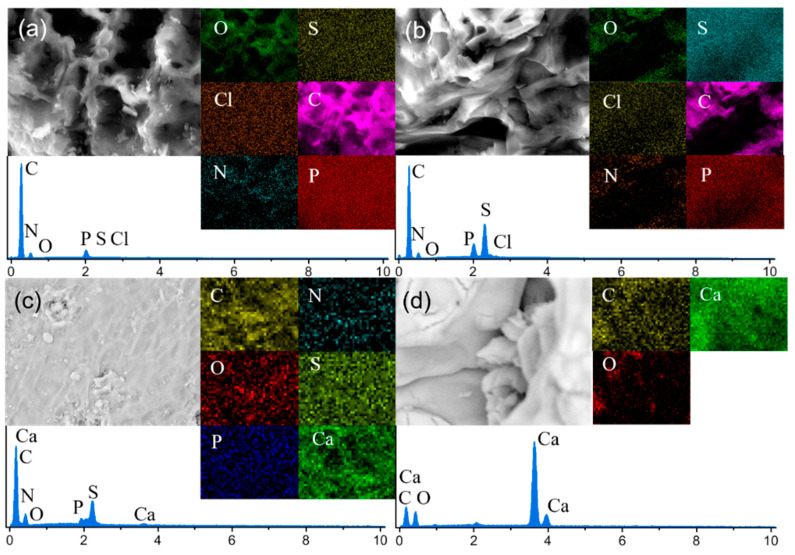
EDS images of (**a**) CN before adsorption, (**b**) CN after adsorption, (**c**) MCS3-1 after adsorption, and (**d**) SS.

The EDS plots and the element content of CN before and after adsorption, MCS3-1 after adsorption, and SS are shown in Figure 4 and Table 1, respectively. The results reveal that P existed in CN. This indicates that the use of phosphoric acid was effective in preparing an activator for activated carbon. In addition, the percentages of N, S, and Cl in CN were greatly increased after adsorption, as shown in Table 1. As MB is composed of C, N, S, and Cl, it was adsorbed in the CN. The reductions in the percentages of O and P resulted from the increases in the percentages of other elements brought about by MB. In contrast to the CN adsorption results, the presence of elemental Ca on the adsorbent surface after the adsorption of MCS3-1 was consistent with the disappearance of the small pore structure analyzed above. Furthermore, the EDS results showed a large increase in both N and O elements in the MCS3-1 sample owing to the significant enhancement of CN adsorption in the presence of SS. The content of these elements was increased by the deposition of MB and SS on the CN surface. The SEM image in Figure 4d shows the SS sample. There were three elements in SS, which were the same as those in CaCO_3_. However, as shown by the results of the TGA-DSC, the main component of the material was calcium oxide, which was different from the ratio of the elements in the EDS results. This indicated that the SS material contained a small amount of CaCO_3_ in addition to CaO.

#### 3.1.4. BET Analysis

**Figure 5:**
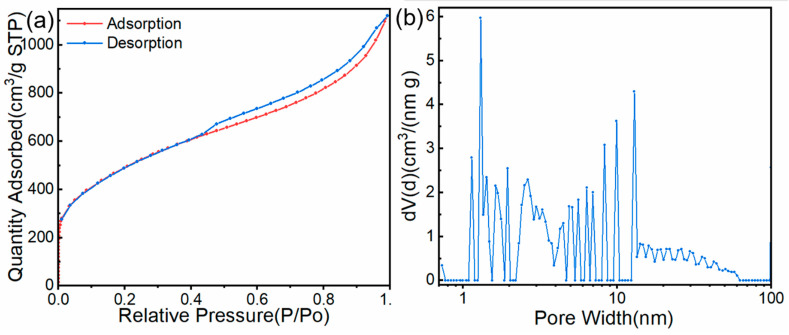
(**a**) Nitrogen adsorption–desorption isotherm and (**b**) pore size distribution of CN.

The SS component of the adsorbent was used for pH adjustment and photocatalysis while CN was used for adsorption, and only CN was investigated using BET analysis. The results are shown in Figure 5a,b. Based on the IUPAC classification, the CN curve is a type IV isotherm with evident H3 hysteresis loops (0.5 < P/P_0_ < 1.0). This indicates that there were lamellar aggregates that produced narrow mesoporous or microporous slits on the surface of the material. The hysteresis loop isotherms have no obvious saturation adsorption plateaus and show a very irregular pore structure. In addition, an adsorption capacity of about 600 mg/g was obtained for MB. A significant increase in the sorption volume was observed when the relative pressure increased from 0 to 0.01 due to the capillary aggregation effect of the micropores [39]. This result was consistent with the pore size distribution as shown in Figure 5b, and the micropores and mesopores were the main pore structures. The specific surface area of CN was 1705 m^2^/g, the pore volume was 1.734 cm^3^/g, and the average pore diameter was 4.069 nm. The specific surface area, pore volume, and pore width increased significantly compared to the non-chemically activated group. The above results showed that, at high temperatures, H_3_PO_4_ could effectively improve the specific surface area and pore structure of biochar produced from chestnut shells. On the other hand, the specific surface area of SS was small at 2.0 m^2^/g and the pore volume was 0.013 cm^3^/g, with a 26 nm pore size. SS was mainly used to increase the pH.

#### 3.1.5. FTIR Analysis

**Figure 6:**
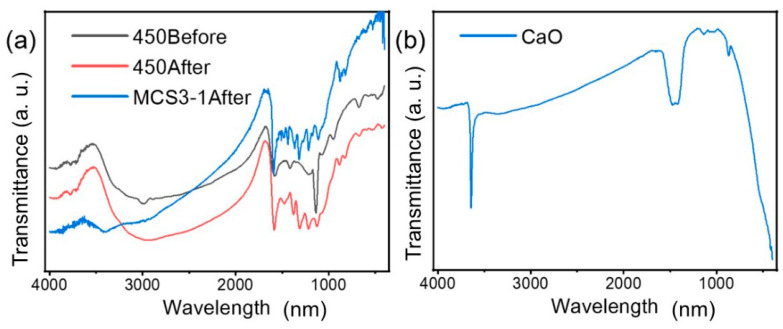
FTIR spectra of (**a**) CN before and after adsorption and (**b**) CaO.

Figure 6a shows the FTIR spectra for CN before and after adsorption and for MCS3-1 after adsorption. They reflect the changes in functional groups and chemical bonds of the adsorbent. Before adsorption, the presence of peaks at 2993 cm^−1^ represented the stretching vibration of C-H on the benzene ring. The peak at 1583 cm^−1^ could be assigned to the aromatic ring in CN. The peaks observed at 1413 and 1217 cm^−1^ were those of -CH_2_ and -CH_3_, respectively. Two other peaks were observed at 1138 and 1074 cm^−1^ belonging to C=O and C-O-C [39], which were amorphous carbon linkage bonds. After adsorption, the intensity of the peak at 1585 cm^−1^ increased significantly, which was due to the aromatic structure in MB, which enhanced the aromaticity after adsorption [40]. This meant that the MB was adsorbed by the adsorbent.

In Figure 6a, partial similarities in the FTIR spectra of CN and MCS3-1 after the adsorption can be observed. The peak around 3000 cm^−1^ represented the stretching vibration of C-H on the benzene ring. In the range from 1600 to 1000 cm^−1^, similar peaks, such as the peak at 1583 cm^−1^, belonged to the C=C stretching vibration. The peaks at 1413 and 1217 cm^−1^ belonged to -CH_2_ and -CH_3_ and other peaks at 1138 and 1074 cm^−1^ belonged to C=O and C-O-C [40]. These identical bonds were due to the presence of the same component (CN) and its adsorption of MB. The small peaks near these bonds were due to the photocatalytic degradation of MB by the SS component in MCS3-1. It broke some of the chemical bonds in MB and caused a tiny shift in the original functional groups.

Figure 6b shows a sharp absorption band for O-H stretching attached to calcium atoms that appeared around 3641 cm^−1^. The following insignificant peak around 3300 cm^−1^ was a vibration of H-O-H due to the H_2_O molecules adsorbed from the air [41]. The peaks at 1475, 1421, 1136, and 867 cm^−1^ were due to the C-O bond related to the carbonation of the sample during the pyrolysis process [42]. The strong band for Ca-O appeared around 500 cm^−1^, corresponding to the Ca-O prepared from calcined snail shells.

#### 3.1.6. XPS Analysis

**Figure 7:**
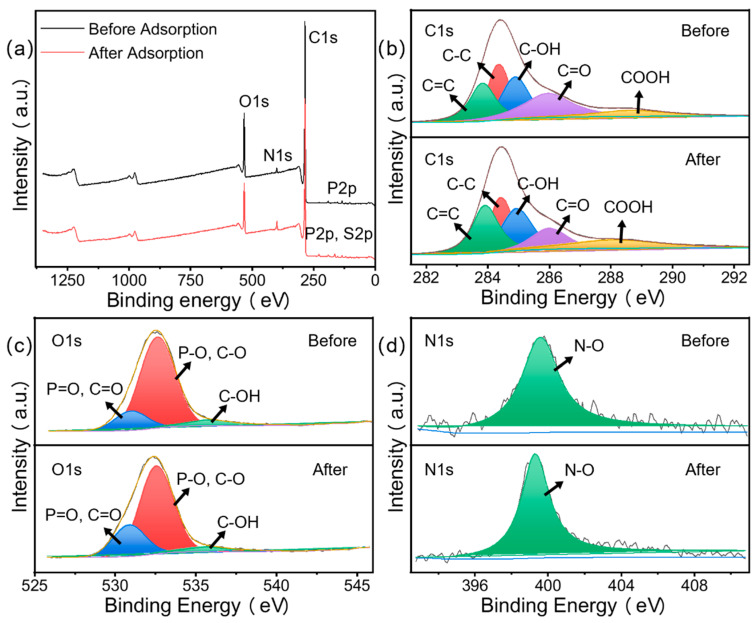
XPS full spectra of (**a**) CN, (**b**) C 1s, (**c**) O 1s, and (**d**) N 1s.

The changes in the content of major elements (C, N, and O) before and after adsorption were analyzed using XPS [34]. The presence of O and N was due to the incomplete carbonization of carbohydrates in the biomass, while the presence of P was due to the chemical pretreatment with phosphoric acid to dope the P during the carbonization process. Figure 7 shows the XPS images of CN before and after adsorption. The increase in the intensity of the N peak observed at 401.2 eV was due to an increase in the elemental N content caused by the adsorption of large amounts of MB by the CN, which is consistent with the EDS results in Section 3.1.3.

In Figure 7b, there are five peaks in the spectrum of C 1s corresponding to the hybridized carbon atoms (C=C, C-C), hydroxyl (C-OH), carbonyl (C=O), and carboxyl (-COOH) in aromatic ring, with variations including shifts of 283.8 to 284.4 eV, 284.3 to 284.9 eV, 283.2 to 283.8 eV, 285.1 to 286.0 eV, and 287.7 to 288.4 eV [43], respectively. The large variation in the binding energy indicates a strong affinity between the dye and the oxygen-containing functional group, suggesting that the latter dominates the whole adsorption process. The O 1s spectrum could be fitted to three peaks corresponding to C=O/P=O (531.2 eV), C-O/P-O (532.7 eV), and C-OH (536.0 eV), which indicates the presence of hydroxyl, carboxyl, and phosphate groups in CN. After MB adsorption on the adsorbent, these peaks shifted to 530.9, 532.6, and 535.7 eV, respectively. This suggests that these chemical bonds are involved in the adsorption process. The N 1s spectrum shows another N-O group shifted from 399.6 to 399.3 eV, accompanied by a large increase in intensity, due to adsorbed MB.

**Figure 8:**
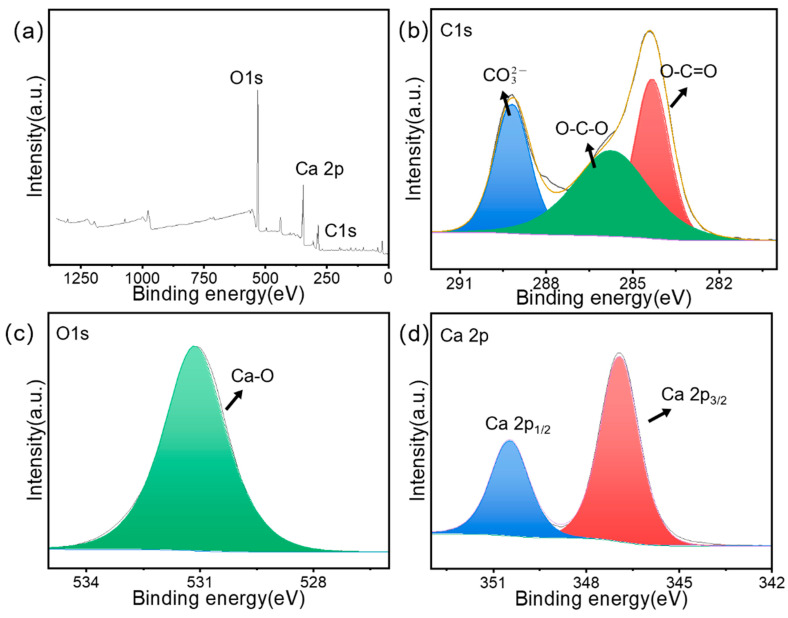
XPS full spectra of (**a**) raw snail shells, (**b**) C 1s, (**c**) O 1s, and (**d**) Ca 2p.

**Figure 9:**
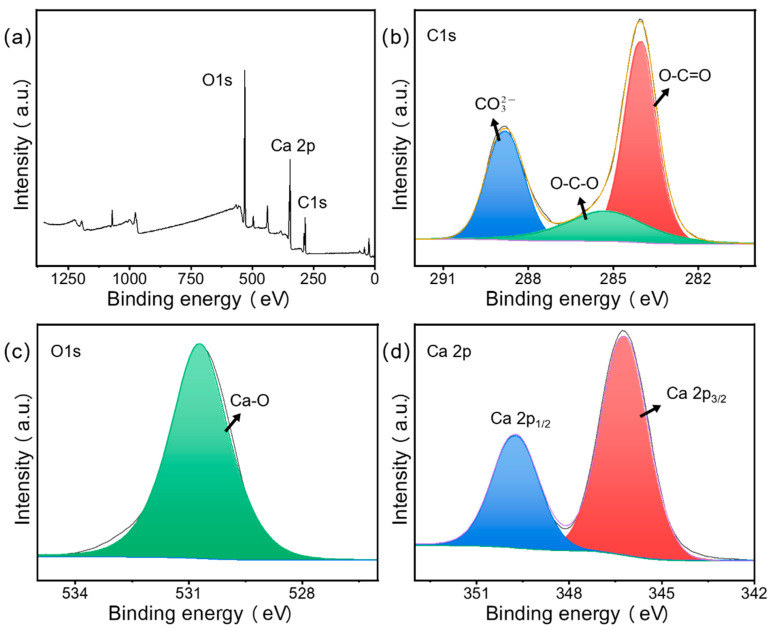
XPS full spectra of (**a**) SS, (**b**) C 1s, (**c**) O 1s, and (**d**) Ca 2p.

The XPS spectra of the raw snail shells and SS samples are shown in Figure 8 and Figure 9. They show the presence of Ca, C, and O elements in the SS sample. The C 1s spectrum (Figure 9b) was decomposed into three peaks at 284.1, 285.3, and 288.8 eV, which were attributed to O-C=O, O-C-O, and CO32- [44]. The O 1s XPS spectrum (Figure 9c) presented a peak at 530.7 eV, which was attributed to the Ca-O bond [45]. The XPS spectrum of Ca 2p had two peaks at 347.2 and 350.7 eV (Figure 9d), which belonged to Ca 2p_3/2_ and Ca 2p_1/2_ [44], respectively. This proved that not only calcium oxide but also some undecomposed calcium carbonate was present in the SS material, which was consistent with the EDS results. Compared to raw snail shells, the strength of O-C-O was significantly reduced due to the transformation of carbon and oxygen to calcium oxide in the material under the high-temperature pyrolysis conditions. In the full spectrum, at 50–200, the raw snail shells group had several spurious peaks, while the SS group was smoother, which was due to the removal of some impurities by the pyrolysis process.

**Figure 10:**
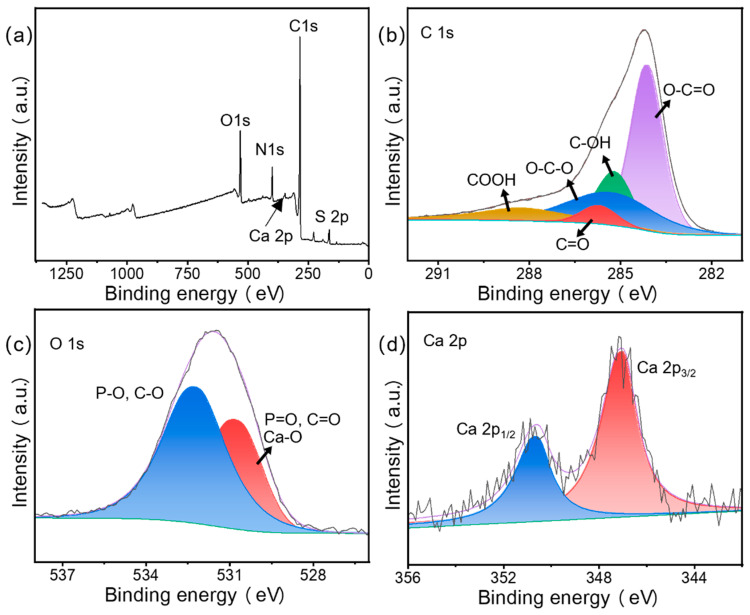
XPS full spectra of (**a**) MCS3-1 after adsorption and photocatalysis, (**b**) C 1s, (**c**) O 1s, and (**d**) Ca 2p.

The XPS spectra for MCS3-1 after adsorption and photocatalysis are shown in Figure 10, with the XPS full spectrum of MCS3-1 shown in Figure 10a. In addition to the elements contained in CN, it also contained Ca, which originated from the calcium hydroxide deposited on the surface of the CN sample. Comparing Figure 9a and Figure 10a, the intensity of the Ca peak decreased dramatically because only a small amount of SS in the mixed sample MCS3-1 was attached to the CN surface. This proved that the 3:1 ratio of the mixture was appropriate, would not cause the SS to block the pore structure of CN, and would result in a reduction in the adsorption capacity. This result was also consistent with the results for C 1s and O 1s. Figure 10b,c show C 1s (b) and O 1s (c). The O-C-O appeared in the C 1s spectrum of MCS3-1 (b) and Ca-O peaks appeared in the O1s spectrum of MCS3-1 (c), both of which were derived from the SS component [44]. Figure 10d shows the clear Ca 2p spectrum indicating the existence of Ca.

### 3.2. Adsorption Experiments

#### 3.2.1. ZETA Potential and Effect of pH

**Figure 11:**
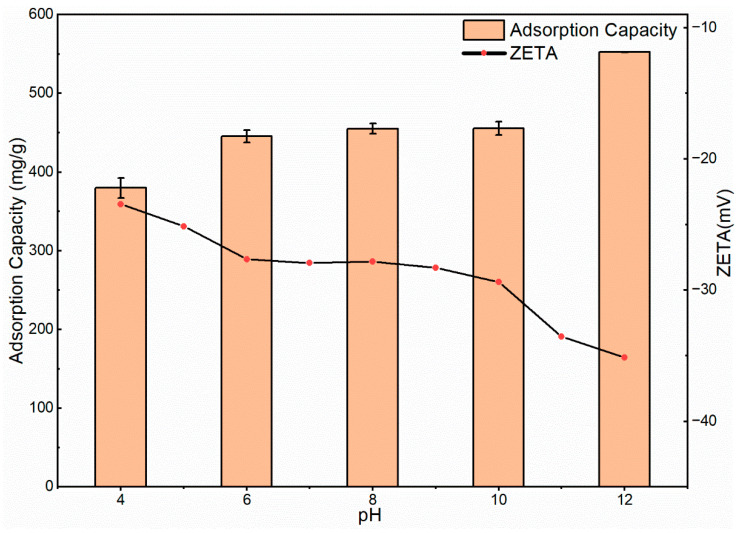
Zeta potential and adsorption of CN at different pH levels.

Next, Since the SS component itself had a role in regulating the pH, only CN samples were tested to study the influence of pH on adsorption capacity and zeta potential. Due to the functional groups and surface charges, such as –OH and –COOH, being affected by pH, it was determined that the initial pH value had a crucial effect on adsorption [46]. The zeta potential was measured directly with the zeta potential instrument and the adsorption capacity of CN was measured at different pH values from 4 to 12; the initial concentration of the MB was 552 mg/L. Figure 11 shows the zeta potential and adsorption of CN at different pH values. The zeta potential values were all negative, which indicated that the CN surface was rich in hydroxyl carboxyl functional groups, and with the increase in the pH, the value of the zeta potential kept decreasing, which meant that more functional groups were exposed on the surface material, and this had a facilitating effect on the adsorption process. The point for zero charges could not appear at pH values greater than 4; therefore, at the pH used, the surface charge was negative.

This was the opposite of the change in adsorption capacity for different pH values because the number of H^+^ ion-binding sites above the CN surface was finite as they were in an acidic environment [47]. At pH levels from 6 to 10, the zeta potential value remained stable, and the surface charge of the material was relatively stable; therefore, the adsorption capacity was similar. After the pH was increased to 12, the potential difference between the dye and the adsorbent was enhanced and the adsorption capacity reached 550 mg/g, which represented a 99.9% removal rate. As MB is a kind of cationic dye, the electric charge has a great influence on the adsorption capacity of the adsorbent. When the absolute value of the zeta potential was quite low, the surface of the adsorbent was in a low potential state due to protonation [48]. At higher pH values, the surface potential of the adsorbent became a high negative potential due to the protons. The aqueous solution of MB provided a positive charge to the system, which led to an electrostatic attraction between the system and the negatively charged adsorbent on the surface, resulting in the excellent adsorption of CN at high pH [12].

In previous studies, chestnut shell-activated carbon (CN) was obtained as neutral activated carbon after washing it with deionized water in the preparation process. For the SS fraction with the pH adjustment effect, the pH of the solution was brought to about 11.2 by adding 5 mg of the sample in 20 mL. This was close to the adsorption conditions for the maximum adsorption capacity of CN, and there was only a small amount of solid precipitation in the solution. When the amount of SS added continued to increase, the pH of the solution increased a little and the precipitation in the solution increased a lot. This was the reason why we chose 5 mg SS with 15 mg CN as the adsorbent in the subsequent experiment.

#### 3.2.2. Effect of Adsorbent Ratio

**Figure 12:**
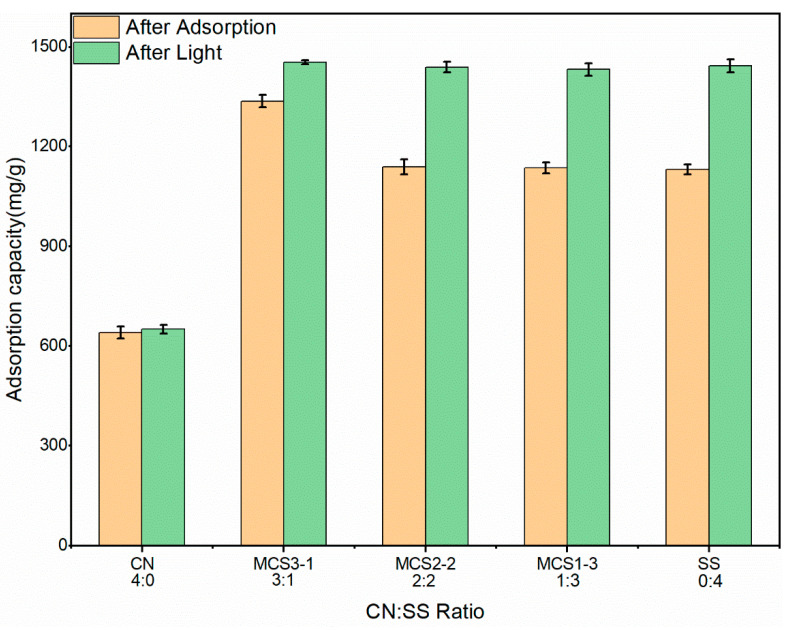
The influence of the adsorbent CN:SS ratio.

The influence of the adsorbent CN:SS ratio is shown in Figure 12. The CN:SS ratios were 4:0, 3:1; 2:2; 1:3, and 0:4 for each adsorbent, respectively, with 20 mg dosage. Due to the great enhancement obtained in the adsorption capacity from increasing pH, an initial concentration for the MB in the ratio experiment of 1476 mg/L was selected to fully demonstrate the adsorption capacities of different adsorbents. The result clearly showed that MCS3-1 had the best adsorption capacity at about 1336 mg/g, with only 640 mg/g in the CN group and 1130 mg/g in the other groups. Although the amount of CN used was higher than in the other groups, the adsorption capacity was lower due to the improved adsorption of CN by the SS component in the mixture. The main component of SS after the pyrolysis was calcium oxide, which reacted with water and dissolved, as shown by Equation (9):

(9) $${\mathrm{CaO}+H}_{2}{O=\mathrm{Ca}(\mathrm{OH})}_{2}↓$$

In a previous study, an alkaline environment improved the adsorption capacity of an adsorbent [49]. This was because the calcium hydroxide was hydrolyzed in an aqueous solution to produce OH^−^, which provided an alkaline solution environment and affected the pH of the solution, the charge, and the level of electricity in the groups on the surface of the adsorbent [50]. The pH of the solution increased with the increase in the SS ratio, which resulted in the enhancement of the electrostatic attraction between the MB and CN. The adsorption capacity increase in the CN was explained by the large adsorption capacity obtained by consuming less CN in the MCS1-3, MCS2-2, and MCS3-1 samples. In addition, the adsorption of the CN relied on the porous structure and large specific surface area to remove MB. On the other hand, the calcium hydroxide produced the precipitated calcium oxide on the CN porous structure, which resulted in blocked pores, reduced adsorption capacity, and excess calcium oxide in the experiment. The adsorption results of these two groups were the same as the SS results, which why almost the same removal rate of about 77% (1130 mg/g) was obtained for the SS, MCS1-3, and MCS2-2 groups after adsorption. At this stage, the removal of MB was due to the degradation of MB by Ca(OH)_2_ and the photocatalysis due to incomplete shading in the experimental equipment.

After the adsorption stage, the solution was placed in an incubator simulating daylight for 24 h to evaluate the photocatalytic performance of CaO. The CN sample lacked photocatalytic ability and the removal rate slightly increased after the exposure to light due to the increase in the adsorption time of the MB. For the other groups, the removal capacity reached 1430 mg/g after the exposure to light and indicated good photocatalytic degradation performance for the SS components. The increase in the CN percentage improved the adsorption capacity of the material because CaO precipitated in the liquid by generating Ca(OH)_2_ that precipitated on the CN surface. It covered the material pores and the adsorption sites were lost. Thus, the removal capacities of these three groups showed very similar characteristics in the degradation of MB by CaO and photocatalysis.

Based on the pH and ratio studies, we chose to use CN and MCS3-1 to remove MB in the aqueous solution in the next experiment.

#### 3.2.3. Effect of Initial MB Concentration and Contact Time

**Figure 13:**
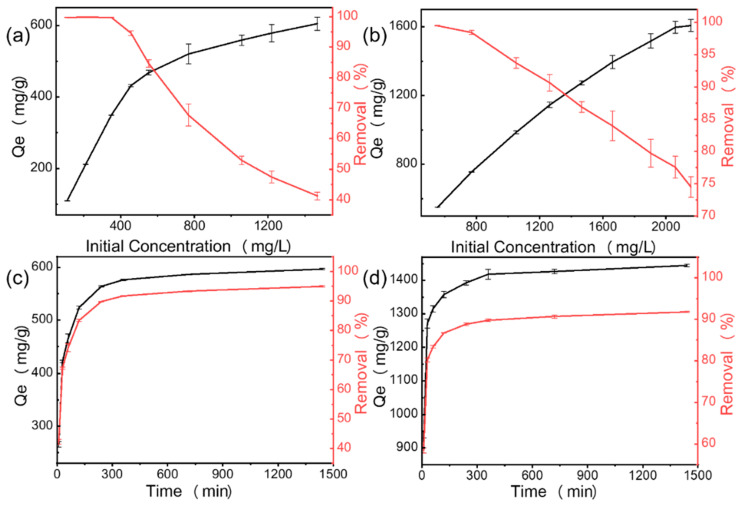
Effect of initial concentration and contact time on adsorption of MB with (**a**,**c**) CN and (**b**,**d**) MCS3-1.

The initial concentration and the contact time were important factors affecting the adsorption capacity of the adsorbent [51]. Figure 13a,b show the effects of the initial concentration on the MB removal efficiency and adsorption capacity for CN and MCS3-1, respectively. In the initial stage of the CN physical adsorption process, a large number of active sites on the surface of the adsorbent promoted rapid adsorption and caused a nearly 100% removal rate at low concentrations. When the initial concentration of MB increased, MB occupied the active sites on the surface of the adsorbent during the adsorption process and the active sites on the surface decreased until adsorption saturation. The saturation led to a reduction in the removal rate from 100% to 40% as the concentration increased. The saturation adsorption amount slightly increased with the increase in the initial concentration, as shown in the curve for CN. However, for the MCS3-1 adsorbent, the amount of adsorption increased due to both physical and chemical adsorption. It was difficult for the degradation processes to reach the equilibrium concentration due to the degradation of MB by the alkaline calcium hydroxide solution. As shown in Figure 13b, the removal rate decreased when the initial concentration increased from 500 ppm to 2100 ppm; however, it was still greater than 75% at 2100 ppm. Therefore, the adsorption capacity and degradation capacity of MCS3-1 were better than those of CN.

Figure 13c,d demonstrate the effect of contact time on adsorption capacity and removal rate. The initial MB concentrations of CN and MCS3-1 were 550 mg/L and 1570 mg/L and the removal rates were 94% and 91%, respectively. The amount of MB adsorbed increased with the increase in adsorption time [52]. The rate of adsorption of MB was fairly rapid at the initial stage in the first 30 min. Then, the adsorption rate slowed from 30 min to 400 min and reached equilibrium after 400 min. The rapid adsorption within 30 min was due to the abundance of unoccupied active sites on the surfaces of MCS3-1 and CN that were accessible for rapid MB attachment. However, the ratio of unoccupied active sites to dye concentration on the material surface decreased. When the adsorption rate decreased, equilibrium was eventually reached. When the concentration increased, the concentration of MB continued to decrease and the removal rate also decreased. However, the adsorption concentration that achieved the 90% removal rate was chosen for comparison since the removal efficiency decreased rapidly.

#### 3.2.4. Adsorption Isotherm

**Figure 14:**
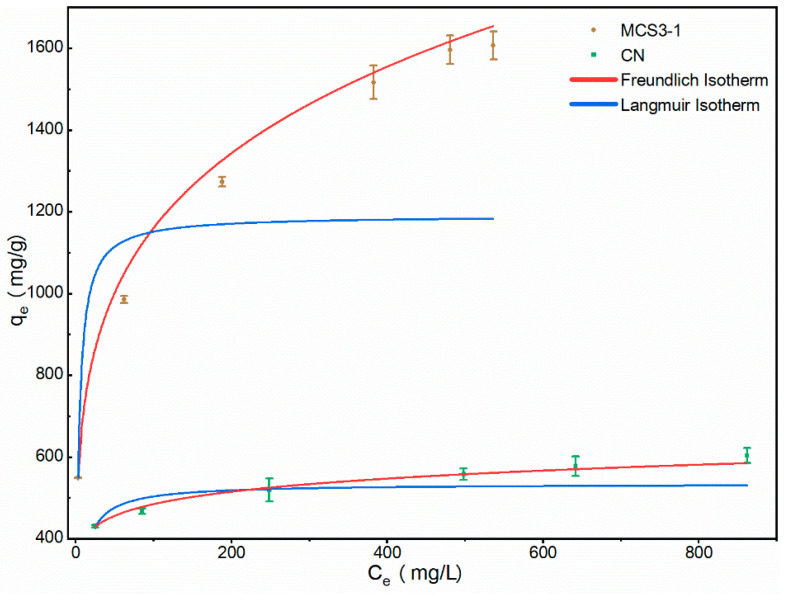
Langmuir and Freundlich isotherm plots of MCS3-1 and CN adsorption of MB.

Figure 14 shows the effect of initial concentration on the adsorption capacity of the adsorbent to further demonstrate the interaction between the adsorbent and MB at the equilibrium state. The sorption isotherms of MB molecules on CN and MCS3-1 at different initial concentrations were measured, and two classical models (Langmuir and Freundlich models) were applied to analyze the experimental data. The correlation coefficients of model predictions versus experimental observations are presented in Figure 14 and Table 2. The Freundlich model was more accurate than the Langmuir model and could effectively predict the sorption isotherm of MB for CN and MCS3-1, implying that MB adsorption with CN and MCS3-1 could be described as multilayer adsorption. The factor 1/n in the Freundlich isotherm model reflected the heterogeneity factor, the heterogeneity of site energies, and the adsorption intensity. Values of 1/n smaller than 0.5 indicated that the adsorbate was easily adsorbed, and values of 1/n larger than 2 indicated that the adsorbate was hardly adsorbed [49,53]. In this study, the values for 1/n for MB adsorption onto both MCS3-1 and CN were less than 0.5, indicating that the adsorbate was easily adsorbed.

The maximum adsorption capacity for MB of CN was 620 mg/g, and the maximum adsorption capacity for MB of MCS3-1 was 1635 mg/g. The results showed that the adsorption capacity of MCS3-1, which was almost three times that of CN, was better than that of CN.

#### 3.2.5. Adsorption Kinetics

The adsorption kinetics were investigated to study the adsorption rate and whether the adsorption process was controlled by chemical or physical adsorption [6]. The equilibrium contact time of the adsorbent was affected by the physical structure and chemical properties. The kinetic investigation involved a systematic method of determining the rate at which contaminants were best removed from an aqueous solution [30]. The adsorption rates of MB dyes on different materials were evaluated using pseudo-first-order (PFO) and pseudo-second-order (PSO) models.

**Figure 15:**
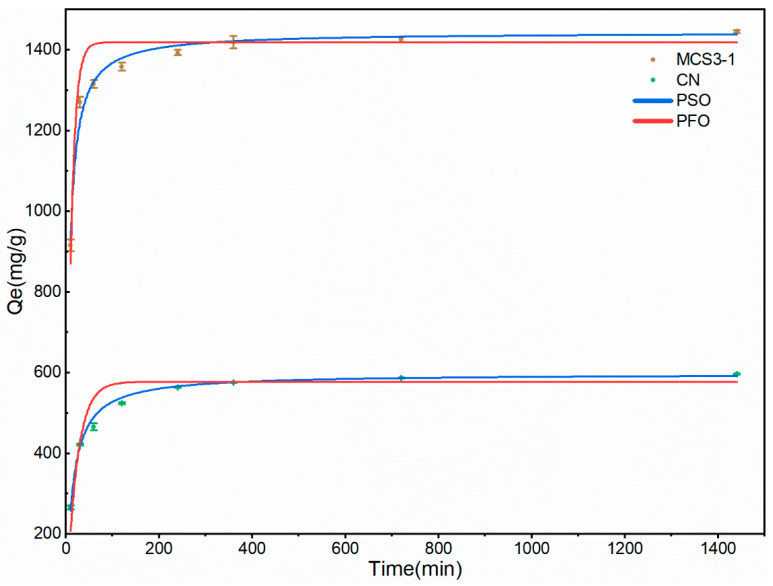
Adsorption kinetics of MCS3-1 and CN for MB.

According to Figure 15 and Table 3, the adsorption equilibrium time reached 400 min, while the fast adsorption process was 90 min. In addition, the equilibrium adsorption amounts of MCS3-1 and CN were 1443.3 and 596.9 mg/g, respectively. The fitted results of the PSO models for both samples had higher correlation coefficients (R^2^) than the PFO models, indicating that the rate-limiting step in the adsorption process was chemisorption, involving chemical interactions between MB and polar functional groups on the surface of the MCS3-1 and CN.

#### 3.2.6. Mechanism of MB Adsorption and Photocatalysis

**Figure 16:**
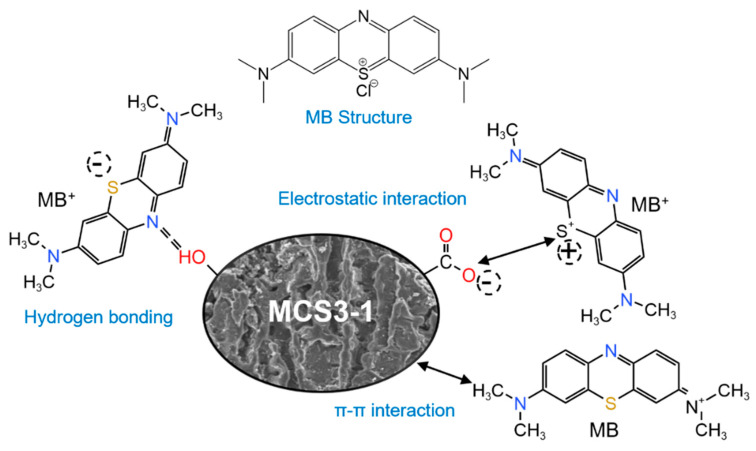
Structure of MB and mechanism of CN adsorption of MB.

The adsorption of MB onto CN was a complicated process in which various interactions coexisted. These included π–π interactions, hydrogen-bond interactions, ion exchange processes, and electrostatic interactions, as shown in Figure 16.

The XPS and FTIR results for SS showed that the main component was CaO, which, as demonstrated by the effects of the pH and zeta potential, could be dissolved in water and decrease the pH; the adsorption mechanism was referred to as electrostatic interaction. The zeta potential decreased with increasing pH, resulted in a negative charge on the surface of CN, and facilitated the adsorption of cationic MB dyes. Therefore, the electrostatic interactions between the CN and MB molecules were important in the adsorption process [6]. In addition, based on the reduction in the C-OH peak in the XPS curves of MCS3-1 and CN, during the adsorption process, the MB molecule electrostatically interacted with the -OH functional group and the active site on the CN surface was first occupied by MB. After the active sites on the surface were filled, MB molecules reached the inner surface of CN through the pore structure and combined with the internal groups and, finally, the adsorption saturation was reached [54]. The π–π interaction between the dye molecules and CN was thought to be a primary driving force facilitating the adsorption of organic pollutants, with the π–π interaction being due to the aromatic structure of MB and the partially graphitized structure in CN. MB molecules had three aromatic rings along with delocalized π electrons and a cationic center, whereas adsorbent CN had many discrete domains of graphitic carbon enriched with π electrons, as well as several structural defects. The π–π interaction caused the adsorption of MB molecules on the CN surface, which resulted in a shift in the position of the peak before and after adsorption in the FTIR images due to conjugation, and more small peaks appeared on the FTIR curve of MCS3-1, which was brought about by the degradation of MB molecules.

The photocatalytic process could be described by the following steps: after obtaining enough energy under light, electrons were excited, jumped from the valence band to the conduction band, and formed holes in the valence band. An electron was obtained from electron donors (H_2_O), resulting in the formation of ·OH free radicals. After that, the MB molecules reacted with the reactive groups through ·OH and O_2_^−^, which eventually led to the decolorization of MB molecules. The specific reaction steps were as follows [25]:

MB + hv → MB * (absorption of photons and excitation of MB molecules);
hv + CaO → e^−^(CB) + H *(VB) (electron–hole pair formation);
e^−^(CB) + O_2_ → ·O_2_^−^ (oxygen ion sorption);
2O_2_^−^ + 2H_2_O → H_2_O_2_ + 2OH^−^ + O_2_ (·O_2_^−^ neutralization by protons);
H_2_O_2_ + e^−^(CB) → -OH + ·OH (decomposition of H_2_O_2_ and ·OH formation);
H^+^ + H_2_O → H^+^ + ·OH (water splitting by photo-hole to produce ·OH radicals);
·OH/·O_2_^−^+ ·MB → MB degradation (electrophilic attack on MB molecules).

Based on the above steps, the degradation of MB molecules started with the cleavage of the double bonds at the R-S^+^=R functional group into R-S(=O)-R and, further, the ·OH/O_2_^−^ radical caused the sequential degradation from R-SO_2_-R and R-SO_3_H-R to the final products SO_4_^2−^, CO_2_, and phenol.

#### 3.2.7. Photocatalysis after Adsorption

**Figure 17:**
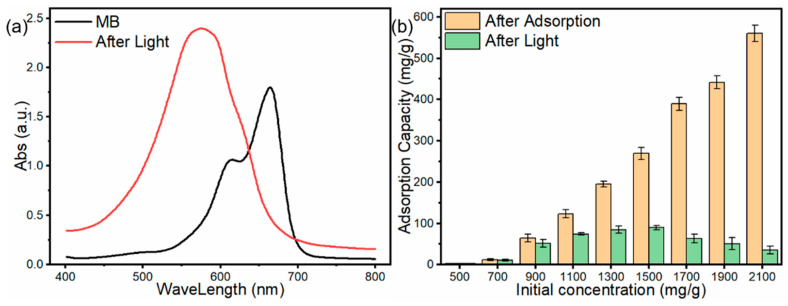
(**a**) UV spectra of MB before and after light exposure, (**b**) MB removal capacity at the different initial concentrations in the solution after adsorption and photocatalytic capacity after light exposure.

The UV spectra and photocatalytic capacities after adsorption are shown in Figure 17. This figure illustrates the variation in MB in an aqueous solution and the shift in the MB peak from 665 nm toward 570 nm. There was no peak shift in the CN sample, which indicated that the changes in the UV spectrum were caused by the SS component and by the degradation and photocatalytic conversion of MB to other substances. The SS component was effective for MB removal. The MB concentration in solution after adsorption and photocatalytic capacity after light exposure is shown in Figure 17b. As the initial concentration increased, the concentration of the solution after adsorption also increased, compounding the Freundlich model. The photocatalytic capacity increased and then decreased with the increase in the light concentration before light exposure. In the beginning, the low photocatalytic capacity was due to the low concentration of MB, which ended the photocatalytic reaction quickly. When the concentration increased, the photocatalytic reaction was not limited by the amount of MB and started to rise; however, with the increase in concentration, there was also a decrease in the light transmission of the solution. Thus, the light entering the liquid decreased due to photocatalysis and not due to a lack of light. Therefore, the photocatalytic capacity decreased in the light phase. In this study, the maximum photocatalytic capacity was in the range of MB concentrations from 180 to 280 mg/L, for which the SS component of the material could exert the maximum photocatalytic capacity of 89 mg/g.

#### 3.2.8. Desorption Experiments

**Figure 18:**
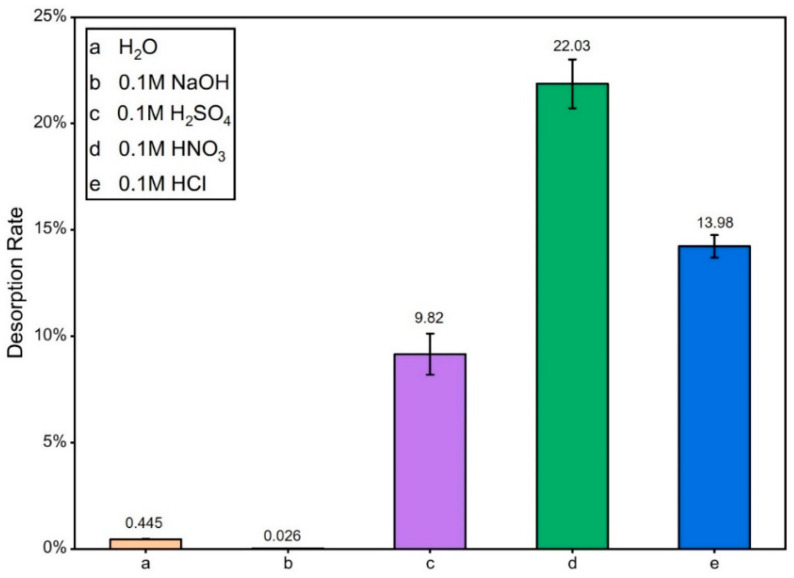
Desorption rate of MCS3-1.

The desorption test is an important indicator of material reuse performance [35]. Figure 18 depicts the desorption of MCS3-1 by different adsorbents. MCS3-1 completed the adsorption process and the content was 1154.2 mg/g. The desorption rate performance was the poorest in pure water and alkaline conditions because, in both neutral and higher alkaline conditions, zeta potentials remained low and negative, as shown in the previous zeta potential studies. When the adsorption of cationic dyes from MB and the adsorbent was completed easily, the desorption was unfavorable. In contrast, when using acidic adsorbent, there was a significant increase in desorption, with highest desorption rates being 22% in the HNO_3_ group and 9.82% and 13.98% in the remaining H_2_SO_4_ and HCl groups, respectively. The low desorption rate was not due to insufficient desorption but to the degradation of MB by the SS component in MCS3-1, which shifted the maximum absorption peak. Even when the desorption was complete, the concentration did not reach the initial level. As the SS results of the proportional study from Figure 12 showed, the degradation of MB by SS in a dark environment reached up to 70%. Therefore, the MCS3-1 material was more easily desorbed in the acidic system, and HNO_3_ worked best as the adsorbent.

## 4. Conclusions

The pollution from the organic dye methylene blue is increasing year by year and, at the same time, there is huge waste from cheap and easily available biomass, such as chestnut shells and snail shells. Therefore, we produced CN with a combined chemical-pyrolysis method and SS with a pyrolysis method. Further, the obtained phosphoric acid-activated chestnut shells were simply mixed with high-temperature pyrolysis snail shell materials. The obtained materials showed high adsorption capacities and photocatalytic degradation in MB solution. Pretreatment with 40% H_3_PO_4_ solution improved the efficiency of MB removal by chestnut shell-activated carbon, which was due to the increase in specific surface area and pore space during pyrolysis. In addition, the pyrolyzed snail shells could enhance the adsorption capacity of CN by providing an alkaline environment, and SS itself showed a degree of photocatalytic degradation capacity, which improved the removal of MB from the aqueous system.

The experiment results showed that the best adsorption efficiency for the mixed adsorbent was achieved when the ratio of CN/SS was 3:1. The adsorption equilibrium data for CN and MCS3-1 samples in the isothermal adsorption curve study could be fitted with Freundlich isotherms, and the maximum adsorption of MB by CN and MCS3-1 was 620 mg/g and 1635 mg/g, respectively. Meanwhile, the adsorption kinetic curves of both samples were well-fitted to pseudo-second-order models, and equilibrium was reached at about 400 min, indicating that the adsorption was multilayer chemisorption. After light exposure, the maximum photocatalytic capacity of MCS3-1 was about 89 mg/g.

The study provides a proven method for the utilization of food waste such as chestnut shells and snail shells, allowing chestnut shell biochar and snail shell pyrolysis products to be prepared from existing and renewable biomass sources with a simple process. Moreover, with excellent adsorption capacity and MB dye degradation, the results open a new avenue for the use of this newly developed hybrid adsorbent in the restoration of polluted aquatic environments.

## Figures and Tables

**Table 1:** Elemental content of CN before and after adsorption, MCS3-1 after adsorption, and SS.

Element	Atomic %			
	CN before Adsorption	CN after Adsorption	SS	MCS3-1 after Adsorption
C	84.81	80.94	35.63	68.19
N	0.00	3.30	-	14.89
O	12.64	7.16	42.31	14.47
P	2.48	2.18	-	0.41
S	0.05	6.20	-	1.84
Ca	-	-	22.05	0.19

**Table 2:** Isotherm parameters of MCS3-1 and CN adsorption of MB.

Isotherms	Parameters	Adsorbents	
		MSC3-1	CN
Langmuir	K_L_ (L/mg)	0.29 ± 0.05	0.16 ± 0.05
	q_m_ (mg/g)	1191.1 ± 98.5	534.7 ± 25.8
	R^2^	0.918	0.767
Freundlich	1/n	0.211 ± 0.008	0.087 ± 0.006
	K_F_ ((mg/g) (L/mg)1/n)	439.3 ± 4.3	325.4 ± 7.5
	R^2^	0.983	0.980

**Table 3:** Kinetic parameters of MCS3-1 and CN adsorption of MB.

Kinetic Models	Parameters	Adsorbents	
		MSC3-1	CN
Pseudo-first-order	k_1_ (min^−1^)	0.095 ± 0.016	0.045 ± 0.007
	q_e_ (mg/g)	1418.5 ± 16.2	557.2 ± 7.4
	R^2^	0.827	0.872
Pseudo-second-order	k_1_ (×10^−4^) (g·mg^−1^·min^−1^)	1.25 ± 0.11	1.28 ± 0.08
	q_e_ (mg/g)	1443.3 ± 6.6	596.9 ± 2.4
	R^2^	0.977	0.992

## Data Availability

The authors confirm that the findings of this study are available within the article.
